# Northern Hemisphere Glaciation during the Globally Warm Early Late Pliocene

**DOI:** 10.1371/journal.pone.0081508

**Published:** 2013-12-12

**Authors:** Stijn De Schepper, Jeroen Groeneveld, B. David A Naafs, Cédéric Van Renterghem, Jan Hennissen, Martin J. Head, Stephen Louwye, Karl Fabian

**Affiliations:** 1 Department of Earth Science, University of Bergen, Bergen, Norway; 2 Geosciences Department, University of Bremen, Bremen, Germany; 3 MARUM – Center for Marine Environmental Sciences, University of Bremen, Bremen, Germany; 4 Alfred Wegener Institute for Polar and Marine Research, Bremerhaven, Germany; 5 Research Unit Palaeontology, Ghent University, Ghent, Belgium; 6 Department of Earth Sciences, University of Toronto, Toronto, Ontario, Canada; 7 Department of Earth Sciences, Brock University, St. Catharines, Ontario, Canada; 8 Norwegian Geological Survey, Trondheim, Norway; University of Oxford, United Kingdom

## Abstract

The early Late Pliocene (3.6 to ∼3.0 million years ago) is the last extended interval in Earth's history when atmospheric CO_2_ concentrations were comparable to today's and global climate was warmer. Yet a severe global glaciation during marine isotope stage (MIS) M2 interrupted this phase of global warmth ∼3.30 million years ago, and is seen as a premature attempt of the climate system to establish an ice-age world. Here we propose a conceptual model for the glaciation and deglaciation of MIS M2 based on geochemical and palynological records from five marine sediment cores along a Caribbean to eastern North Atlantic transect. Our records show that increased Pacific-to-Atlantic flow via the Central American Seaway weakened the North Atlantic Current and attendant northward heat transport prior to MIS M2. The consequent cooling of the northern high latitude oceans permitted expansion of the continental ice sheets during MIS M2, despite near-modern atmospheric CO_2_ concentrations. Sea level drop during this glaciation halted the inflow of Pacific water to the Atlantic via the Central American Seaway, allowing the build-up of a Caribbean Warm Pool. Once this warm pool was large enough, the Gulf Stream–North Atlantic Current system was reinvigorated, leading to significant northward heat transport that terminated the glaciation. Before and after MIS M2, heat transport via the North Atlantic Current was crucial in maintaining warm climates comparable to those predicted for the end of this century.

## Introduction

**Figure 1:**
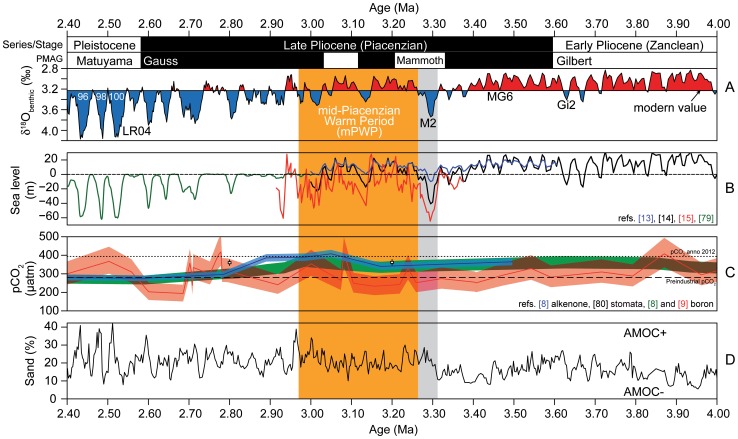
Marine isotope stage M2 in the long-term climate evolution of the Pliocene. (A) Time scale, including palaeomagnetic reversals (PMAG) and the LR04 benthic isotope stack [10], orange shading shows mid-Piacenzian Warm Period ( =  mid-Pliocene Warm Period), grey shading shows marine isotope stage MIS M2; (B) sea level estimates for the Pliocene to Pleistocene [13]–[15], [79]; (C) Late Pliocene atmospheric carbon dioxide concentrations based on boron, alkenones and leaf stomata [8], [9], [80]; (D) long-term carbonate-sand record at ODP Site 999 as an indicator for Pacific water flow through the Central American Seaway into the Atlantic and AMOC [27].

**Figure 2:**
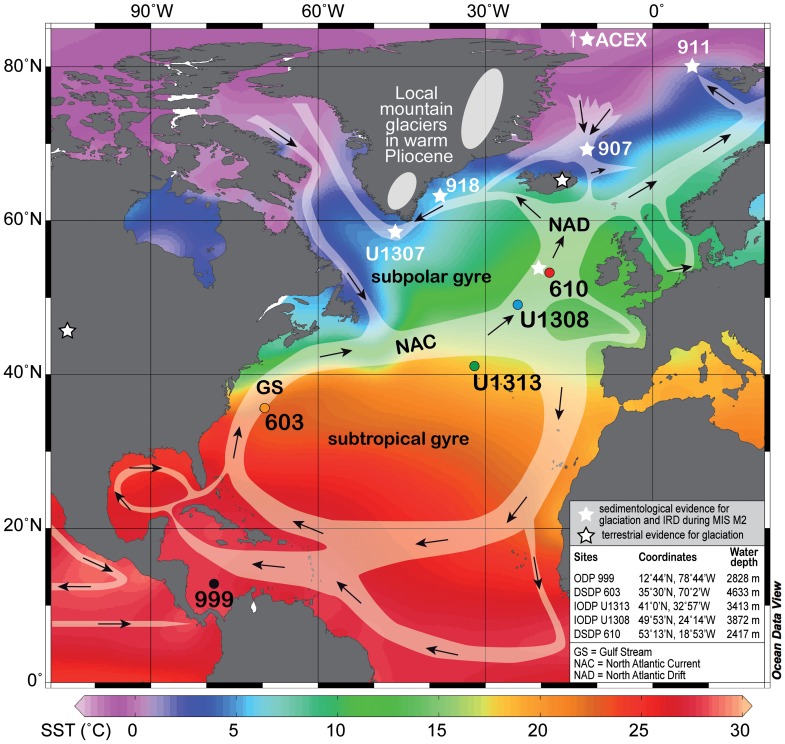
Modern North Atlantic surface circulation with modern sea surface temperatures (World Ocean Atlas 2005 [81]). Each studied site is indicated by the same colour in subsequent figures, and other sites discussed in the text are shown in white. Ice caps on Greenland are schematic representations of Pliocene reconstructions [4].

The early Late Pliocene (early Piacenzian) from 3.6 to ∼3.0 million years ago (Ma) is the last sustained interval in Earth's history when global climate was warmer than today. The ∼3.3–3.0 Ma time slab known as the mid-Piacenzian Warm Period (mPWP, Figure 1) has been studied intensively as a potential analogue for our future global climate [1]. The mPWP is characterised by ∼3°C warmer global temperatures [2], 10–40 m higher sea-level [3], reduced continental ice sheets [4], and an Atlantic meridional overturning circulation (AMOC) comparable to [5] or stronger than [6] preindustrial levels. Atmospheric CO_2_ concentrations were higher than preindustrial values, and likely as high as the modern anthropogenic values of ∼400 ppm [7]–[9] (Figure 1C). The mPWP climate is a good approximation for the warm climatic conditions of the entire early Late Pliocene. This warm stable climate was nonetheless interrupted by a short-lived, intense global glaciation (3.305–3.285 Ma) during marine isotope stage (MIS) M2 [10], [11] (Figure 1). In the LR04 Plio-Pleistocene benthic δ^18^O stack [10], MIS M2 starts as a low-amplitude glaciation typical of the Pliocene, but deepens steeply between 3.305 and 3.285 Ma to reach values characteristic of early Quaternary glaciations. We distinguish this brief interval of intense glaciation (3.305–3.285 Ma) within the longer interval of MIS M2 (3.312–3.264 Ma) as defined in LR04 [10]. The associated glacio-eustatic sea level drop is reflected in a major depositional sequence boundary [12] with sea level estimated at 10 m±10–15 m, 40 m±10 m, or indeed up to 65 m±15–25 m below present [13]–[15] (Figure 1B). Given this large uncertainty in reconstructed sea level for MIS M2, it is difficult to quantify how the volume of the northern and southern hemisphere ice sheets changed. Using the Holocene-like, relatively cool and dry Arctic climate at Lake El'gygytgyn (northeast Arctic Russia) as an approximation of the broader Arctic climate, ice advance during MIS M2 is thought to have occurred in Alaska, Greenland, Svalbard and Antarctica, whereas substantial expansion in North America was less likely [16]. Estimates for ice volume increase in Antarctica correspond to a sea level drop of ∼8 m [17] or even ∼18 m [13], but cannot not fully explain the ∼0.5‰ benthic foraminiferal δ^18^O shift at this time [10]. Direct and indirect evidence of glaciation support expansion of the Antarctic ice sheet [18], [19], a considerable ice advance of the Greenland and Svalbard/Barents Sea ice sheets [20]–[23], ice cap expansion in Iceland [24], and possibly in Alaska and the Canadian Rocky Mountains [25] (Figure 2).

Interrupting an interval of global warmth, MIS M2 has been proposed as an early, failed attempt by the Earth's climate to establish a pattern of intense and frequent Northern Hemisphere glaciations [26], [27]. It was not until ∼500,000 years later that this pattern emerged, likely due to decreasing atmospheric carbon dioxide concentrations during the Late Pliocene [8], [28]. The decline in atmospheric carbon dioxide concentrations [7]–[9], increasing global ice volume [10], [11], cooling of ocean surface waters [29]–[31], and tectonic closure of ocean gateways [27], [32] since the Late Miocene may well have ultimately facilitated glaciation in the late Late Pliocene, but these long-term processes are an unlikely cause of the short-lived MIS M2 glaciation. Similarly, variations in astronomical forcing alone cannot explain the intense glaciation of MIS M2 because intervals with similar astronomical forcing occurred throughout the Late Pliocene without leading to intense glaciation. The isolated nature of the MIS M2 glaciation in the otherwise warm climate of the early Late Pliocene must be the result of a specific forcing, unique within this time period.

We established high-resolution palynological and geochemical records from five ocean drilling sites along a southwest–northeast transect in the North Atlantic covering the Caribbean Warm Pool, Gulf Stream, subtropical gyre and North Atlantic Current (NAC) over the interval 3.400–3.180 Ma to determine the role of ocean circulation in causing the extensive glaciation of MIS M2 (Figure 2). Our surface water mass, sea surface temperature (SST), relative salinity reconstructions, and carbonate-sand records provide direct evidence that the unique conditions responsible for glaciation during MIS M2 relate to an increased Pacific-to-Atlantic flow via the Central American Seaway (CAS) prior to MIS M2. This weakened northward heat transport due to a shift of the NAC. The conceptual model proposed here links an open CAS with glaciation in the Northern Hemisphere and contrasts with hypotheses that propose the closure of the CAS as a cause for the intensification of Northern Hemisphere glaciation around 2.6 Ma [33], or as a delaying factor [34] or a precondition for ice sheet expansion in the Northern Hemisphere [27].

## Materials and Methods

Samples were collected at the IODP Bremen Core Repository (Germany) and Gulf Coast Repository (College Station, Texas, USA) from five sites constituting a transect between the Caribbean Sea (ODP Site 999), western North Atlantic (DSDP Site 603), and the eastern North Atlantic (DSDP Site 610, IODP Sites U1308 and U1313). The foraminiferal geochemistry data and palynomorph assemblages were acquired from the same samples at each of the five ocean drilling sites, and samples for biomarker (alkenone) analysis were taken from the same sample depths at three sites. All generated dinoflagellate cyst and geochemical proxy data are accessible through the database PANGAEA at http://doi.pangaea.de/10.1594/PANGAEA.804677. Previously published Mg/Ca and dinoflagellate cyst data [26], [35] are also available at http://doi.pangaea.de/10.1594/PANGAEA.758710 and http://doi.pangaea.de/10.1594/PANGAEA.758711.

### Dinoflagellate cyst preparation technique and assemblage interpretation

**Figure 3:**
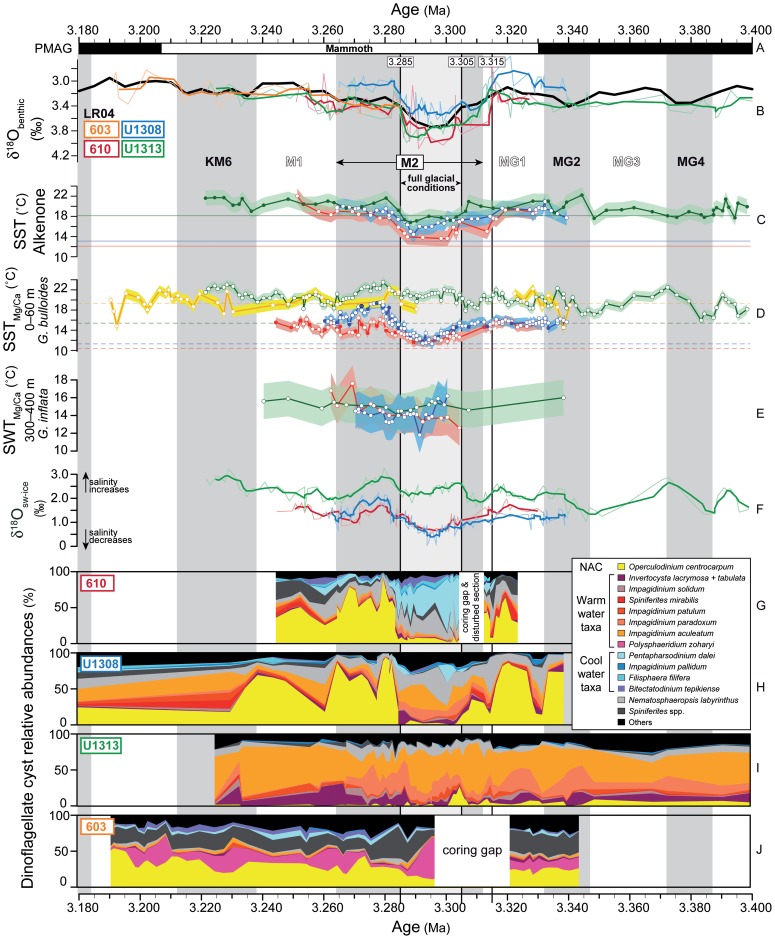
North Atlantic palaeoceanographic proxy records from DSDP Sites 603 and 610, and IODP Sites U1308 and U1313 between 3.400 and 3.180 Ma. Circles with white fill are data points from this study, circles with colour fill are from [30], [55] (C) and [26], [35] (D). Vertical grey bars represent glacials, white bars are interglacials. (A) palaeomagnetic reversals; (B) benthic isotope age models for each site tuned to the LR04 stack [10] (black line), thin coloured lines are data, thick coloured line is 4-point running mean; (C) alkenone SSTs (0–10 m water depth) including calibration related error (shading), horizontal lines represent modern average annual temperature at 0–50 m water depth for each site; (D) SSTs at 0–60 m water depth based on Mg/Ca of *G. bulloides*, including a 1°C error bar (shading), horizontal lines represent modern average spring (March-April-May) temperature at 0–50 m water depth for each site; (E) SWT_Mg/Ca_ of *G. inflata* at 300–400 m water depth; (F) calculated δ^18^O_sw-ice_ as estimate of salinity; thick coloured lines are a 4-point running mean; (G–J) dinoflagellate cyst assemblage composition. High abundances of *O*. *centrocarpum* (yellow) indicate an active NAC. Bluish colours  =  cool-water species, reddish colours  =  warm-water species; (G) and (H) contain data presented in [26] and [35]. (J) *B. tepikiense* and *F. filifera* are grouped together at DSDP Site 603 and are represented by the colour for *B. tepikiense* (purple); *Impagidinium* cf. *pallidum* is represented as *Impagidinium pallidum*.

Our laboratory technique allows dinoflagellate cysts and foraminifera to be extracted from the same samples (full details in [26]). Each sample was first wet sieved at 125 µm to concentrate the foraminifera and ensure that the palynomorphs pass through the sieve for further processing. The fraction retained on the sieve (>125 µm) was dried and weighed before being picked for foraminifera. The sediment filtrate (<125 µm) was dried and weighed, and *Lycopodium clavatum* tablets were added before applying standard palynological preparation techniques involving cold HCl and HF acids [36]. No oxidation, alkali or ultrasonic treatments were used. Organic residues were sieved through a 10-µm nylon mesh and strew mounted onto microscope slides using glycerine jelly. Dinoflagellate cysts were counted under 400x magnification with counts varying between 44 and 527 (average 267) specimens per sample. In addition, acritarchs and terrestrial palynomorphs were also enumerated during the dinoflagellate cyst counts. Palynomorph concentrations and error estimates were then calculated based on the palynomorph and *Lycopodium clavatum* counts and the dry weight of the <125 µm fraction [37]. Only relative abundance variations that are statistically significant according to the procedure described in ref. [38] have been used for interpretation. Data presented in refs. [26] and [35] were used alongside our newly generated data (Figure 3).

The assemblage composition of dinoflagellate cysts in core-top samples is largely related to the present-day overlying water masses [39]–[42], and reflects the interplay between temperature, salinity, nutrients, sea ice cover and light availability. Present-day, last interglacial [43] and Pliocene [26] dinoflagellate cyst assemblages recovered from the eastern North Atlantic consisting of high abundances of *Operculodinium centrocarpum* sensu Wall & Dale (1966) (herein *O. centrocarpum*) all reflect the presence of the North Atlantic Current (NAC). At DSDP Site 610 and IODP Site U1308, *O. centrocarpum* concentrations are highest also when the relative abundances are high, independently corroborating the value of *O*. *centrocarpum* as a NAC indicator species.

Full authorial citations of species discussed in the text are given in Table 1.

**Table 1:** Dinoflagellate cyst species mentioned in the text and figures: abbreviation, full authorial citation and grouping.

Abbreviation	Full species name
*B. tepikiense*	*Bitectatodinium tepikiense* Wilson 1973
*F. filifera*	*Filisphaera filifera* Bujak 1984 emend. Head 1994
*I. aculeatum*	*Impagidinium aculeatum* (Wall 1967) Lentin & Williams 1981
*I. paradoxum*	*Impagidinium paradoxum* (Wall 1967) Stover & Evitt 1978
*I. pallidum*	*Impagidinium pallidum* Bujak 1984
*I. patulum*	*Impagidinium patulum* (Wall 1967) Stover & Evitt 1978
*I. solidum*	*Impagidinium solidum* Versteegh & Zevenboom in Versteegh 1995
*I. lacrymosa*	*Invertocysta lacrymosa* Edwards 1984
*I. tabulata*	*Invertocysta tabulata* Edwards 1984
*N. labyrinthus*	*Nematosphaeropsis labyrinthus* (Ostenfeld 1903) Reid 1974
*O. centrocarpum*	*Operculodinium centrocarpum* sensu Wall & Dale 1966
*O. israelianum*	*Operculodinium israelianum* (Rossignol 1962) Wall 1967
*P. dalei*	Cyst of *Pentapharsodinium dalei* Indelicato & Loeblich III 1986
*P. zoharyi*	*Polysphaeridium zoharyi* (Rossignol 1962) Bujak et al. 1980
RBC	Round brown cysts
*S. mirabilis*	*Spiniferites mirabilis* (Rossignol 1964) Sarjeant 1970, and
	*Spiniferites hyperacanthus* (Deflandre & Cookson 1955) Cookson & Eisenack 1974
*Spiniferites/ Achomosphaera* spp.	*Spiniferites* spp. Mantell 1850, and
	*Achomosphaera* spp. Evitt 1963
Others	Contains all other dinoflagellate cyst taxa counted.

### Geochemistry: δ^18^O and Mg/Ca of foraminifera, calculating and interpreting sea surface temperature and relative salinity (δ^18^O_sw-ice_)

Foraminifera were picked from the >125 µm dry fraction of each sample. Planktonic foraminifera isotope data were measured using a Finnigan MAT 251 mass spectrometer at the Isotope Laboratory, Geosciences Department, University of Bremen using five specimens per sample of *Globigerina bulloides* (250–315 µm) for Sites 603, 610, U1308 and U1313 and five specimens per sample of *Globigerinoides sacculifer* (250–355 µm) for Site 999. Benthic foraminiferal isotope data are based on at least one >250 µm specimen of *Cibicidoides wuellerstorfi* or *Uvigerina perigrina* per sample. *Cibicidoides wuellerstorfi* δ^18^O values have been corrected by adding 0.64 ‰ [44]. The standard deviation of the analyses is based on an in-house Solnhofen carbonate standard with a value of 0.07‰. Values are reported relative to that of the Vienna Pee Dee Belemnite (VPDB) calibrated using National Bureau of Standards (NBS) 18, 19, and 20 standards.

For Mg/Ca measurements, we used 20–25 specimens per sample of *G. bulloides* (250–315 µm) or *G. sacculifer* (250–355 µm) and 20 specimens per sample of *Globorotalia inflata* (250–400 µm) from Sites 610, U1308 and U1313. The cleaning procedure for Mg/Ca measurements is described elsewhere [45]. After dissolution in 0.5 mL 0.075 M QD HNO_3_, the samples were centrifuged and diluted for analysis on an ICP-OES (Perkin Elmer Optima 3300R) at the Geosciences Department, University of Bremen. The analytical precision of the Mg/Ca analyses for *G*. *bulloides*, *G. sacculifer*, and *G. inflata* combined was 0.17*%* (n = 459). Reproducibility based on replicate samples (n = 32) of both *G. bulloides* and *G. sacculifer* was ±0.11 mmol/mol (∼3.3%). The validity of analyses was checked by analysing an artificial in-house standard to monitor drift of the ICP-OES (Mg/Ca  = 2.93 mmol/mol) and the limestone standard ECRM752-1 (Mg/Ca  = 3.75 mmol/mol) to allow inter-laboratory comparison [46]. Al/Ca, Fe/Ca, and Mn/Ca were simultaneously analysed with Mg/Ca to prevent contaminated samples from being included in the interpretation. We used the following calibration, established from core-top sediment samples in the North Atlantic, to transform the foraminiferal Mg/Ca ratios of *G. bulloides* into SST_Mg/Ca_: Mg/Ca  = 0.52 exp 0.10 T [47]. We interpret the SST_Mg/Ca_ value of *G. bulloides* as spring to summer SSTs of the upper 60 m of the water column [26], [48], [49] because the oxygen isotope composition of *G. bulloides* reflects the northward-migrating phytoplankton spring bloom in the North Atlantic [47], [50]. The SST_Mg/Ca_ value of *G. sacculifer* represents the annual mixed-layer temperature of the upper 75 m of the water column for Caribbean Site 999 [51]. Mg/Ca values were transformed into palaeo-seawater temperatures using the following equation: Mg/Ca  = 0.491 exp 0.033 T [52]. Although *G. inflata* calcifies throughout the water column, the SWT_Mg/Ca_ based on mostly non-encrusted *G. inflata* represents the temperature of the permanent thermocline [53]. We used the following calibration to calculate temperatures: Mg/Ca  = 0.72 exp 0.076 T. Combining analytical and calibration errors, we estimate the error on Mg/Ca palaeotemperature reconstruction for shallow-dwelling foraminifera as ±1.0–1.5°C [47], whereas for the deeper-dwelling *G. inflata* the error is estimated to be ±2–2.5°C [53].

The oxygen isotope composition of seawater (δ^18^O_sw_) was calculated via a standard formula [54]. Since Mg/Ca and δ^18^O were measured on the same planktonic foraminiferal species, the possible effects of seasonality and habitat differences are minimised. We used the LR04 global benthic foraminiferal δ^18^O stack [10] as an approximation for changes in ice volume over the studied interval. After normalizing the LR04 record, we subtracted it from δ^18^O_sw_, resulting in a δ^18^O_sw-ice_ record that approximates local variations in salinity.

### Alkenones

All alkenone data from Sites 610 and 1308 are new, whereas data from IODP Site U1313 have been published earlier [30], [55] (Figure 3C). The modified alkenone unsaturation index 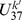 [56], [57] was measured using a GC/TOF-MS system [58] on separate samples, taken from the same depths as those used for foraminiferal Mg/Ca and dinoflagellate cyst analyses. 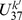 in combination with a global core-top calibration was used to calculate annual mean SST (top 10 m) [59]. The analytical technique, calibration and reliability of alkenone-based SSTs for the Pliocene is detailed elsewhere [30], [55]. The calibration error on the alkenone SSTs is ∼1.5°C [59].

The global core-top calibration gives the highest correlation with annual mean SSTs, but locally alkenone-based SSTs could reflect the temperature of the growing season (spring in the North Atlantic) [60]. Although this affects the absolute SST estimates, it does not influence the relative trends in our records. The exception would be if the alkenone producers shifted their production season on a glacial/interglacial basis, but there is no evidence for such behaviour. However, if such shifts did occur during glacials the alkenone producers would have delayed their production towards summer to avoid the colder spring surface conditions. This implies that the cooling observed in the alkenone records during MIS M2 would actually underestimate the true cooling.

### Carbonate sand fraction

We generated high-resolution carbonate sand fraction data from ODP Hole 999A over the study interval. In addition, we used the available low resolution, long-term carbonate sand fraction record of the same site [27]. The sand content (>63 µm) of deep-sea carbonates is considered [27] a sensitive indicator of changes in carbonate dissolution: sand content (foraminifer tests) decreases as dissolution progresses. A low-carbonate sand fraction was interpreted to reflect a poorly-ventilated deep Caribbean water mass. Carbonate dissolution at Site 999, caused by entry of Antarctic Intermediate Water (AAIW) into the Caribbean Basin in place of North Atlantic Deep Water (NADW), implies an open Central American Seaway and a weak overturning circulation [27].

### Palaeomagnetic measurements

The positions of magnetic reversals for the Mammoth Subchron in DSDP Holes 603C and 610A [61], and IODP Hole 1308C [62] were re-measured in this study to increase precision by analysing discrete, oriented samples at 4–23 cm resolution (Tables 2–4). Oriented cubic polystyrene boxes (7.2 cm^3^) were taken, avoiding visible mineral concretions and areas influenced by the coring process, from the working halves of Sections 603C-17X1 to 603C-18×1 (12 samples between 136.51 and 146.37 mbsf), Sections 610A-17H3 to 610A-17H4 (17 samples between 156.74 and 158.75 mbsf), Sections 610A-17H6 to 610A-18H1 (19 samples between 161.20 and 163.85 mbsf), and Sections U1308C-26H2 to U1308C-26H5 (20 samples between 230.48 and 239.45 mbsf). The discrete samples were measured at the Geosciences Department, University of Bremen on a cryogenic magnetometer (model 2G Enterprises 755 HR). The natural remanent magnetization (NRM) was demagnetized in nine steps (10–100 mT), and inclination and relative declination, and their confidence intervals were determined from line fits of straight-line segments in a Zijderveld diagram. Note that absolute declination depends on section and core orientations, whereas inclination values rely on the fact that the drill hole is to a very good approximation perpendicular. All ages of the magnetic reversals are according to the ATNTS 2004 [63].

**Table 2:** Palaeomagnetic data for the reversal at the base of the Mammoth Subchron in DSDP Hole 603C (depth indicated in bold).

Site	Hole	Core, type		Section	Half	Top (cm)	Bottom (cm)	Depth (mbsf)	Sample name	Inclination (°)	Inc (low)	Inc (high)	Comments
603	C	17	X	1	W	91	93	136.51	C17S1091	−8.4	−20.8	4.8	Questionable reliability
603	C	17	X	2	W	47	49	137.57	C17S2047	−19.3	−43.1	13.2	Questionable reliability
603	C	17	X	3	W	11	13	138.71	C17S3011	−32.9	−35.0	−30.7	
603	C	17	X	3	W	42	44	139.02	C17S3042	−15.4	−18.1	−12.6	
603	C	17	X	3	W	102	104	139.62	C17S3102	−50.0	−51.7	−48.3	
603	C	17	X	3	W	107	109	139.67	C17S3107	−24.7	−29.3	−19.6	
603	C	17	X	4	W	119	121	141.29	C17S4119	−19.7	−34.4	−1.7	Questionable reliability
603	C	17	X	5	W	41	43	142.01	C17S5041	16.0	12.0	19.9	
603	C	17	X	6	W	42	44	143.52	C17S6042	14.8	11.1	18.3	
603	C	18	X	1	W	11	13	**145.31**	C18S1011	−22.9	−33.0	−11.0	
603	C	18	X	1	W	99	101	**146.19**	C18S1099	28.6	27.1	30.1	
603	C	18	X	1	W	117	119	146.37	C18S1117	39.6	36.5	42.5	

The reversal at the top of the Mammoth Subchron could not be assigned.

**Table 3:** Palaeomagnetic data for the reversals at the base and top of the Mammoth Subchron in DSDP Hole 610A.

Site	Hole	Core, type		Section	Half	Top (cm)	Bottom (cm)	Depth (mbsf)	Sample name	Inclination (°)	Inc (low)	Inc (high)	Comments
610	A	17	H	3	W	73	75	156.74	Mam-N1821	41.2	10.6	57.4	
610	A	17	H	3	W	88	90	156.89	Mam-N1822	48.9	36.4	57.2	
610	A	17	H	3	W	98	100	156.99	Mam-N1823	69.6	44.4	77.2	
610	A	17	H	3	W	108	110	157.09	Mam-N1824	62.9	53.9	68.5	
610	A	17	H	3	W	115	117	157.16	Mam-N1825	53	45.9	58.3	
610	A	17	H	3	W	127	129	157.28	Mam-N1826	56.6	50.7	61.1	
610	A	17	H	3	W	139	141	157.40	Mam-N1827	55.4	30.4	66.6	
610	A	17	H	4	W	6	8	157.57	Mam-N1828	51.9	39.2	60	
610	A	17	H	4	W	21	23	157.72	Mam-N1829	42.1	−42.5	69.8	
610	A	17	H	4	W	26	28	157.77	Mam-N1830	45.4	29	55.8	
610	A	17	H	4	W	36	38	157.87	Mam-N1831	49.8	34.3	59.4	
610	A	17	H	4	W	52	54	158.03	Mam-N1832	78.2	71.2	81.5	
610	A	17	H	4	W	69	71	158.20	Mam-N1833	48	40.1	54.1	
610	A	17	H	4	W	84	86	158.35	Mam-N1834	45.3	25	57.3	
610	A	17	H	4	W	92	94	**158.43**	Mam-N1835	22.7	2.5	38.4	
610	A	17	H	4	W	114	116	**158.65**	Mam-N1836	−17.2	−40	12.4	
610	A	17	H	4	W	124	126	158.75	Mam-N1837	−30.7	−41.8	−16.5	
610	A	17	H	6	W	69	71	161.20	Mam-N1838	−66.5	−70	−61.6	
610	A	17	H	6	W	88	90	161.39	Mam-N1839	−58.7	−61.6	−55.3	
610	A	17	H	6	W	93	95	161.44	Mam-N1840	−50.2	−53.7	−46.1	
610	A	17	H	6	W	101	103	161.52	Mam-N1841	−56.8	−58.9	−54.5	
610	A	17	H	6	W	117	119	**161.68**	Mam-N1842	64.1	62.3	65.7	
610	A	17	H	6	W	134	136	**161.85**	Mam-N1843	−39.2	−41	−37.3	
610	A	17	H	CC	W	11	13	**161.97**	Mam-N1844	20.8	10.9	29.6	
610	A	18	H	1	W	2	4	**162.63**	Mam-N1845	44.1	43	45.3	Disturbed?
610	A	18	H	1	W	8	10	**162.69**	Mam-N1846	−3.7	−6.5	−1	Disturbed?
610	A	18	H	1	W	14	16	**162.75**	Mam-N1847	43.4	41.8	45	Disturbed?
610	A	18	H	1	W	23	25	**162.84**	Mam-N1848	7.1	6.1	8.1	Disturbed?
610	A	18	H	1	W	27	29	**162.88**	Mam-N1849	−20.6	−23.9	-17.1	Disturbed?
610	A	18	H	1	W	31	33	**162.92**	Mam-N1850	−71.3	−74.9	−65.6	
610	A	18	H	1	W	41	43	**163.02**	Mam-N1851	−57.1	−59.4	−54.4	
610	A	18	H	1	W	50	52	**163.11**	Mam-N1852	34.2	31	37.3	
610	A	18	H	1	W	68	70	163.29	Mam-N1853	9.7	−8.9	26.6	
610	A	18	H	1	W	86	88	163.47	Mam-N1854	57.3	34.5	67.6	
610	A	18	H	1	W	101	103	163.62	Mam-N1855	37.4	−12.4	60.2	
610	A	18	H	1	W	124	126	163.85	Mam-N1856	17.5	4.7	28.7	

Depths in bold demonstrate the position of the reversals.

–30 cm of Section 610A-18H1. Note: The reversal at the base of the Mammoth Subchron is difficult to identify due to a core gap and potentially disturbed sediments in the upper 0

**Table 4:** Palaeomagnetic data for the reversals at the base and top of the Mammoth Subchron in IODP Hole U1308C.

Site	Hole	Core, type		Section	Half	Top (cm)	Bottom (cm)	Depth (mbsf)	Sample name	Inclination (°)	Inc (low)	Inc (high)	Comments
1308	C	25	H	6	W	7	9	**230.48**	Mam-N1857	44.7	35.1	51.9	
1308	C	25	H	6	W	15	17	**230.56**	Mam-N1858	−38.1	−42.7	−32.8	
1308	C	25	H	6	W	25	27	230.66	Mam-N1859	−76.6	−79.9	−70.5	
1308	C	25	H	6	W	35	37	230.76	Mam-N1860	−62	−64.5	−59.1	
1308	C	25	H	6	W	42	44	230.83	Mam-N1861	−57	−58.6	−55.2	
1308	C	25	H	6	W	48	50	230.89	Mam-N1862	−51.8	−53.6	−49.8	
1308	C	25	H	6	W	56	58	230.97	Mam-N1863	−60.6	−61.5	−59.6	
1308	C	25	H	6	W	65	67	231.06	Mam-N1864	−69	−70.5	−67.3	
1308	C	25	H	6	W	77	79	231.18	Mam-N1865	−54.8	−56.4	−53.1	
1308	C	25	H	6	W	87	89	231.28	Mam-N1866	−72.4	−73.1	−71.6	
1308	C	25	H	6	W	94	96	231.35	Mam-N1867	−67	−67.7	−66.3	
1308	C	26	H	5	W	33	35	238.74	Mam-N1868	−51.7	−52.4	−51	
1308	C	26	H	5	W	40	42	238.81	Mam-N1869	−75.4	−76.1	−74.6	
1308	C	26	H	5	W	48	50	238.89	Mam-N1870	−54.8	−56.7	−52.8	
1308	C	26	H	5	W	56	58	238.97	Mam-N1871	−57.9	−61	−54.2	
1308	C	26	H	5	W	63	65	239.04	Mam-N1872	−66.5	−69	−63.5	
1308	C	26	H	5	W	72	74	239.13	Mam-N1873	13	−31.1	46.8	
1308	C	26	H	5	W	83	85	**239.24**	Mam-N1874	−42.9	−54.4	−24.8	
1308	C	26	H	5	W	94	96	**239.35**	Mam-N1875	61	0.6	74.4	
1308	C	26	H	5	W	104	106	239.45	Mam-N1876	65.6	62.9	67.9	

Depths in bold demonstrate the position of the reversals.

In DSDP Hole 610A, the upper boundary of the Mammoth Subchron was found between 158.35 mbsf (positive inclination) and 158.75 mbsf (negative inclination). The reversal at the base of the Mammoth Subchron is more difficult to identify due to a coring gap between Cores 610A-17H and 610A-18H, and disturbed sediment in the upper 25 cm of Section 610A-18H1 [64], but must be located between 161.85 mbsf (negative inclination) and 163.11 mbsf (positive inclination). The reversals bounding the Mammoth Subchron in IODP Site U1308 are between 254.56 and 255.46 mcd at the top, and between 262.91 and 264.41 mcd at the bottom [62]. Our re-assessment places the top of the subchron between 254.59 and 254.67 mcd, and the bottom between 263.21 and 263.46 mcd. The offset between predicted and measured position in IODP Site U1308 is small and considered to be within the margin of accuracy of the methods. At IODP Site U1313, the reported palaeomagnetic reversal of the base of the Mammoth Subchron lies at 153.68 mcd ±0.1 m [65], within the glacial maximum of MIS M2. The offset may be the result of the field geometry and distance between the sites, variation in the magnetic lock-in time and depth in the sediment, and the sites used to determine the Mammoth Subchron in the Geomagnetic Polarity Time Scale.

### Age models

An age model was established for each hole (Figures S1–S4) by tuning its benthic foraminiferal stable oxygen isotope record to the LR04 benthic foraminiferal isotope stack [10], with the palaeomagnetic reversals as guidelines only, using the software program AnalySeries 2.0.4.2 [66]. The accuracy of each age model depends on the accuracy of the LR04 benthic stack which is estimated at 15 kyr between 3 and 4 Ma [10] and the accuracy of the graphic correlation of the benthic δ18O records with the LR04 tuning target. The tie-points used for the age models of each hole and their correlation coefficients are presented in Tables 5–9 and Figures S1–S5. The ages in the Results and Discussion section are reported with high precision (3 decimals) to demonstrate (1) the relative age difference between samples within one site, and (2) the relative age of events in relation to the onset (∼3.315 Ma), full glaciation (∼3.305–3.285 Ma), and termination of MIS M2 (∼3.285 Ma). These ages should neither be considered as absolute ages, nor as evidence for suborbital age control.

**Table 5:** Tie-points for the age model of DSDP Hole 610A.

Depth (mbsf)	Age (Ma)
158.60	3.207
159.00	3.233
159.32	3.237
159.90	3.253
160.20	3.263
161.27	3.285
161.56	3.290
161.75	3.301
163.05	3.315
163.89	3.326
164.09	3.332
167.08	3.596

=  metres below sea floor. Note: mbsf

**Table 6:** Tie-points for the age model of IODP Hole U1308C.

Depth (mcd)	Age (Ma)
254.66	3.207
259.27	3.265
261.11	3.284
262.35	3.302
263.25	3.320
263.38	3.327
264.70	3.340

=  metres composite depth. Note: mcd

**Table 7:** Tie-points for the age model of IODP Site U1313 (primary splice).

Depth (mcd)	Age (Ma)
149.92	3.224
150.83	3.237
153.07	3.285
154.39	3.311
157.23	3.372
159.72	3.419

=  metres composite depth. Note: mcd

**Table 8:** Tie-points for the age model of DSDP Hole 603C.

Depth (mbsf)	Age (Ma)
136.70	3.194
138.25	3.214
139.79	3.237
140.27	3.252
142.85	3.286
145.71	3.330

=  metres below sea floor. Note: mbsf

**Table 9:** Tie-points for the age model of ODP Site 999.

Old age (Ma)	New age (Ma)
3.205	3.205
3.239	3.245
3.276	3.280
3.296	3.295
3.319	3.320
3.342	3.340
3.355	3.365
3.371	3.375

[78], new age from this study. Note: old age from ref.

The original age model of ODP Site 999 [27] is based on correlating the benthic δ^18^O record to the astronomically dated benthic δ^18^O records from equatorial East Pacific ODP Site 846 [67] and equatorial East Atlantic ODP Site 659 [68] for the time interval 5–2 Ma. The existing age model was updated [51] to the newly generated, orbitally-tuned age model of East Pacific IODP Site 1241 [69] (Figure S5). For this study, we used the LR04 benthic δ^18^O stack [10] to fine-tune the glacial-interglacial transitions around MIS M2.

## Results and Discussion

### Events during interglacial MIS MG1 leading to glaciation

**Figure 4:**
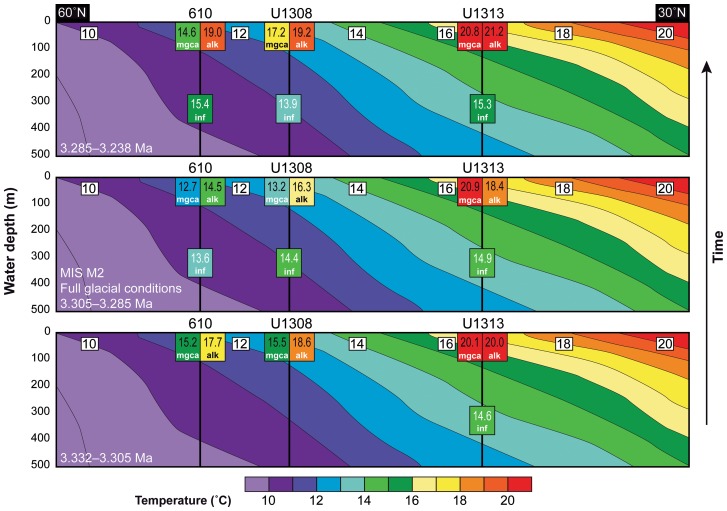
Reconstructed sea-surface temperatures for the studied intervals prior to, during and after the full glacial conditions of MIS M2 along an eastern North Atlantic transect through DSDP Site 610, and IODP Sites U1308 and U1313 from 60°N, 10°W to 30°N, 30°W. Background represents present day sea-water temperatures of the upper 500 m (from WOA2005, [81]). The insets below each site represent the surface water temperature based on alkenones (alk; surface water 0–10 m), Mg/Ca ratios of *Globigerina bulloides* (mgca; mixed layer 0–60 m) and sea-water temperature at 300–400 m based on Mg/Ca ratios of *Globorotalia inflata* (inf). The surface waters (SST_alk_, SST_Mg/Ca_ *G. bulloides*) show important cooling during MIS M2 with a steep N–S temperature gradient established. Low resolution sea-water temperature (SWT_Mg/Ca_) reconstructions of *Globorotalia inflata* show that water at the permanent thermocline (300–400 m depth) remained stable and warm throughout the entire studied period. The glacial had no major effect on the deeper surface waters, except possibly at Site U1308 where one sample recorded temperatures as low as 11.8°C during MIS M2. The average values of 13.5, 14.2 and 15.1°C at Site 610, U1308 and U1313 respectively over the entire period illustrate that the entire upper water column during the Pliocene was warmer than today in the North Atlantic.

The early Late Pliocene was warmer than today, and prior to ∼3.315 Ma our geochemical proxies and dinoflagellate cyst assemblages demonstrate a surface circulation comparable to today's but with elevated temperatures in the high-latitude North Atlantic. We record an active Gulf Stream over Site 603 as illustrated by the high SSTs (ca. 19.5°C) and the presence of *O. centrocarpum* and such warm water dinoflagellate cyst taxa as *Impagidinium aculeatum*, *I. paradoxum*, *I. patulum*, *I. solidum*, and *Polysphaeridium. zoharyi* which are also present there today [41]. Warm (ca. 20°C) and oligotrophic surface waters at the subtropical gyre Site U1313 are reflected in the dominance of *I. aculeatum*, *I. paradoxum, I. patulum*, and *Invertocysta* spp. An active NAC brought warm waters (15.2–18.6°C in the uppermost 60 m) northward over Sites U1308 and 610 (Figure 3C, 3D, 3G, 3H), expressed in the dinoflagellate cyst assemblages by the dominance of *O. centrocarpum* and the persistent presence of the warm water species *Spiniferites mirabilis*. The less steep meridional SST gradient compared to present, especially visible in the SST_alk_ and to lesser extent in the SST_Mg/Ca_ (Figure 3C, 3D, 4), indicates generally warmer conditions in the higher latitudes compared to today.

**Figure 5:**
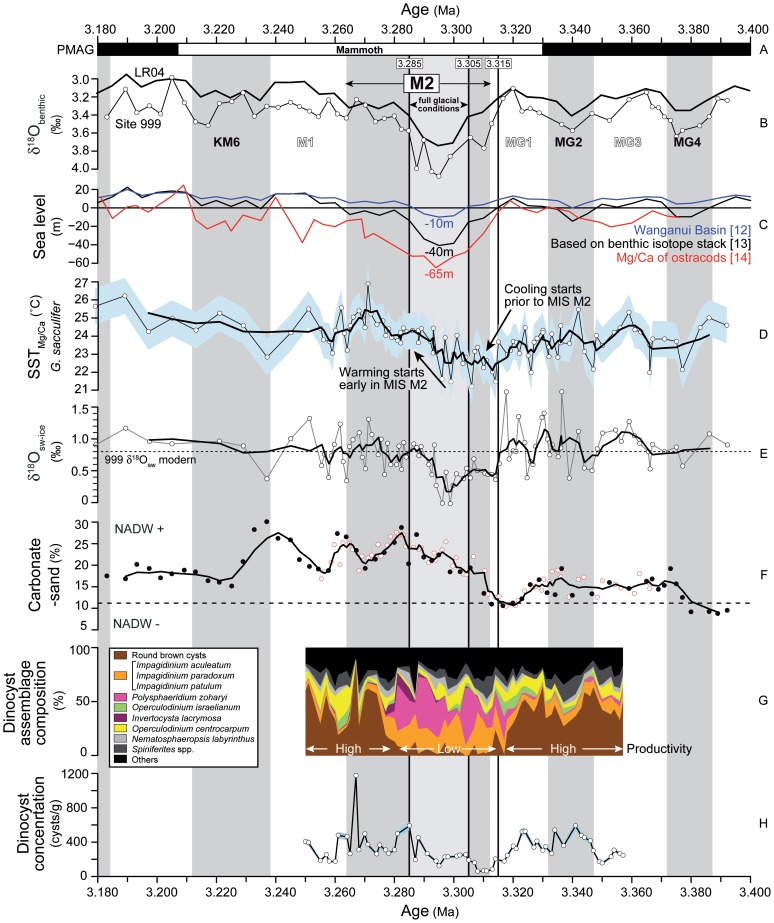
Caribbean Sea palaeoceanographic proxy records from ODP Site 999 between 3.400 and 3.180 Ma. Vertical grey bars represent glacials, vertical white bars are interglacials. All circles with white fill are data from this study. (A) Palaeomagnetic reversals; (B) benthic isotope age model for Site 999 tuned to the LR04 stack [10] (black line); (C) sea level estimates; (D) SST­_Mg/Ca_ of *G. sacculifer*, thick black line represents 4-point running mean, shading represents calibration error; (E) δ^18^O_sw-ice_ estimate of salinity; (F) carbonate-sand fraction, filled black circles are data from [27], thick line represents 4-point running mean. Lower (higher) values reflect decreased (in­creased) North Atlantic Deep Water (NADW) influence at the site. (G) dinoflagellate cyst assemblage composition; presence of round brown cysts indicate high productivity and inflow of Pacific water; (H) dinoflagellate cyst concentration, including error bar (light blue shading).

Although deeper-water exchange via the CAS had been restricted since ∼4.6 Ma [27], shallow Pacific-to-Atlantic exchange occurred well into the Late Pliocene [51]. This implies that Atlantic meridional overturning circulation (AMOC) [6] was able to function even when the CAS was partially open. Following a maximum in AMOC due to minimal Pacific-to-Atlantic through-flow around 3.6 Ma, a gradual increase in through-flow via an open CAS culminated immediately prior to MIS M2 [27], [51]. At Caribbean Site 999, we record between ∼3.320 and ∼3.315 Ma a drop in SST and salinity (δ^18^O_sw-ice_) (Figure 5D, 5E), a low carbonate sand-fraction (Figure 5F), and high productivity evidenced by high dinoflagellate cyst concentrations dominated by heterotrophic species (round brown cysts; Figure 5G, 5H). The low carbonate sand-fraction indicates a poorly ventilated deep Caribbean water mass and carbonate dissolution caused by entry of Antarctic Intermediate Water (AAIW) into the Caribbean Basin in favour of North Atlantic Deep Water (NADW) – interpreted as evidence of a weak overturning circulation [27]. The drop in SST and salinity point to an increased inflow of cooler, less saline Pacific waters to the Caribbean. Furthermore, the inferred high productivity is fully consistent with nutrient-rich waters from the Pacific entering the Caribbean [70]. Considered altogether, this evidence shows that Pacific-to-Atlantic through-flow via the CAS during interglacial MIS MG1 exceeded a critical threshold, thereby reducing the AMOC. This was likely aided by the high sea levels at that time [12], [15] and a longer-term gradual weakening of the thermohaline circulation since 3.6 Ma [27] that brought the climate system closer to a tipping point. Prior to MIS MG1 (Figure 1D, 5C) high sea levels also occurred, but Pacific-to-Atlantic through-flow appears not to have weakened the NAC, and glaciation in the Northern Hemisphere remained restricted.

During maximal Pacific inflow via the open CAS during interglacial MIS MG1 (∼3.315–3.320 Ma), contemporaneous changes occurred in the North Atlantic surface circulation. At ∼3.315 Ma, a major reduction in northward flow of warm NAC waters is reflected at Sites 610 and U1308 by a major turnover of the dinoflagellate cyst assemblages within 1–2 kyrs, and an initial cooling of the surface waters is registered (Figure 3C, 3D, 3G, 3H). This corroborates modelling studies showing that an open CAS results in a weakened AMOC and hence northward heat transport [71], [72]. Between ∼3.315 and 3.305 Ma, a persistent NAC influence is recorded at Site U1308 when SSTs decreased further and *O. centrocarpum* (our NAC tracer) remained present in low abundance. At the same time we find a peak abundance of *O. centrocarpum* (Figure 3I) and surface-water cooling (Figure 3D) at subtropical gyre Site U1313. We interpret this sequence of events as an initial reduction in northward flowing warm water of the NAC at ∼3.315 Ma, followed by a gradual southward deflection of the NAC between ∼3.315 and 3.305 Ma. It is important to note that the initial reduction in northward transport of warm NAC water occurred at ∼3.315 Ma within the interglacial MIS MG1, well before the MIS M2 glacial maximum at 3.295 Ma.

The southward shift of the NAC prior to MIS M2 led to the cessation of northward heat transport during the full glacial conditions of MIS M2 (3.305–3.285 Ma). During the glacial conditions, subtropical gyre circulation persisted as attested by a continuance of the Gulf Stream at Site 603 where no major changes in the dinoflagellate cyst assemblages were recorded (Figure 3J). The contrasting proxy evidence of cool surface SST_alk_ [30], warmer mixed-layer SST_Mg/Ca_ and largely unchanged dinoflagellate cyst assemblages at subtropical gyre Site U1313 is difficult to interpret (Figure 3C, 3D, 3I). During the earlier glacials MIS MG4 and MG2, both geochemical proxies record a cooling, suggesting a fundamentally different oceanography for MIS M2. It is not known whether the divergence in SST_Mg/Ca_ values during MIS M2 was caused by different a genotype of *G. bulloides* becoming dominant in a changed oceanographic setting [73]. Irrespective of the ultimate cause, we consider the contrasting SST proxy records in combination with the palynological data as evidence of a southward shift of the NAC that affected Site U1313 during MIS M2 but not during prior glacials.

### Glaciation in the Northern Hemisphere during MIS M2

At the two northern sites, dinoflagellate cyst assemblages indicate subpolar conditions (*Bitectatodinium tepikiense*, *Filisphaera filifera*, *I. pallidum, Nematosphaeropsis labyrinthus*, *Pentapharsodinium dalei*) at Site 610 and oligotrophic conditions (*I. aculeatum*, *I. paradoxum*) at Site U1308 (Figure 3G, 3H), while surface waters at both sites cooled by 3–4°C to temperatures only just higher than today (Figure 3C, 3D). This cooling at the northern sites established a steep latitudinal SST gradient in the North Atlantic (Figure 4), causing the thermal isolation of Greenland from northward heat transport. As a comparison, a 3–4°C cooling of the Nordic Seas was necessary for the last glacial inception (∼115,000 years ago) in Scandinavia [74]. The increased meridional SST gradient will have reduced air temperature and increased snowfall over most of North America, both factors favourable to ice sheet inception [29]. We demonstrate that sufficiently cool surface waters were present in the northern high latitude oceans, and propose that these were crucial for the glaciation in the Northern Hemisphere during MIS M2. The moisture required to build a large ice sheet in the Northern Hemisphere was presumably already present in the atmosphere, because Pliocene climates were generally wetter than today [75]. It is nevertheless likely that after the southward shift of the NAC and cooling of the northern high-latitude surface waters, carbon cycle (vegetation, CO_2_) [28], [71] and perhaps sea ice (albedo) feedbacks also contributed to the major glaciation during MIS M2. Nevertheless, the extent of Northern Hemisphere glaciation during MIS M2 remained smaller than a typical Quaternary glaciation, but may have been larger than at present. The higher δ^18^O_benthic_ values during MIS M2 compared to today (Figure 1A; 3.74‰ vs. 3.23‰, [10]) indeed imply that MIS M2 ice sheets were larger than today. Indirect evidence of expanded ice sheets in the Northern Hemisphere is found in several sediment and ice-rafted debris records from the Arctic Ocean, Nordic Seas and northern North Atlantic [20]–[23] which indicate that the Greenland and Svalbard/Barents Sea ice sheets reached the coastline. Glacial deposits on Iceland [24] and possibly also in the Canadian Rocky Mountains and Alaska [25] demonstrate the presence of ice caps there. With SSTs approaching present day values (Figure 3C, 3D) and a Holocene-like Arctic climate prevailing during MIS M2 [16], the development of a significant North American ice sheet is unlikely. Therefore, to explain the observed ∼0.5‰ benthic isotope shift [10], a considerable expansion of the Antarctic ice sheet must have occurred also [16]. Nevertheless, the possibility of an ice cap in North America during MIS M2 should not be excluded given the evidence of an ice cap in the North American interior that did not reach the North Atlantic coastline at ∼3.5 Ma [76], when glacials (e.g. MIS MG6) were less severe than during MIS M2 (Figure 1).

### Glacial closure of the Central American Seaway led to deglaciation

Our results further demonstrate that the sea level drop at the full glaciation of MIS M2 [12], [15] closed the CAS and effectively halted the inflow of Pacific water into the Atlantic realm. The oligotrophic conditions at Site 999 shown in the absence of heterotrophic dinoflagellate species and low cyst concentrations (Figure 5G, 5H) during MIS M2 suggest no inflow of nutrient-rich Pacific waters [70]. The increasing SST and salinity (δ^18^O_sw-ice_) at Site 999 from the glacial maximum at ∼3.295 Ma onwards show that Caribbean surface waters became warmer and more saline while remaining oligotrophic (Figure 5D, 5E, 5G, 5H). This is a reflection of the build-up of the Caribbean Warm Pool already from the glacial maximum onwards. The expansion and warming of the Caribbean Warm Pool are essential for re-establishing AMOC and northward heat transport, as observed for the last deglaciation [77]. At around 3.285 Ma, Site 999 is characterised by high SSTs and salinity, a return to biologically productive conditions, and increased carbonate preservation (high carbonate sand-fraction) (Figure 5F). This indicates a Caribbean Warm Pool sufficiently large and warm to re-invigorate the AMOC. The re-established northward heat transport and active NAC flowing along its modern pathway around 3.285 Ma is reflected by the rapid turnover within 1–2 kyrs of the dinoflagellate cyst assemblages in the eastern North Atlantic Sites 610 and U1308 where *O. centrocarpum* becomes dominant again (Figure 3G, 3H). By then, the warm climates of the mPWP [1] were established, with North Atlantic SSTs ∼3°C above present values (Figure 3C, 3D), a modern-like AMOC [5] but with a reduced meridional sea-surface temperature gradient (Figure 4), and a Greenland ice sheet that was reduced to isolated mountain glaciers [4]. As such, the glaciation during MIS M2 appears responsible for its own demise.

## Conclusions

**Figure 6:**
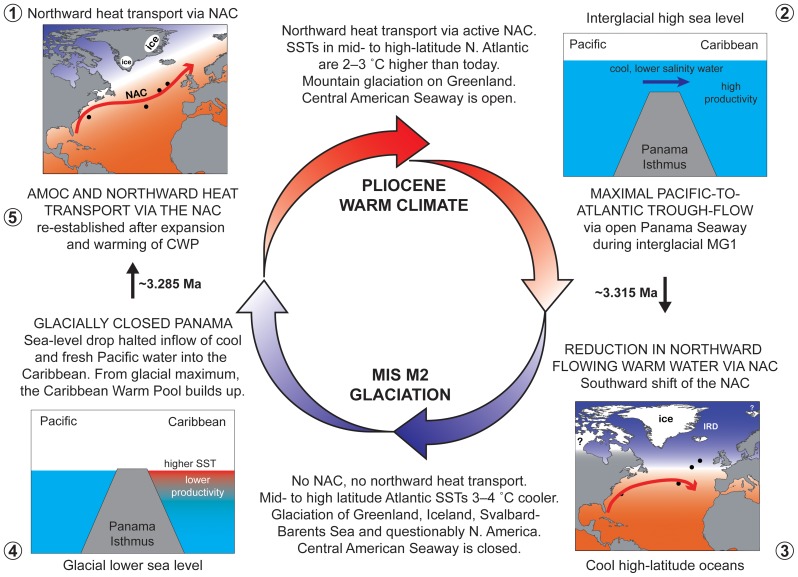
Conceptual model of glaciation and deglaciation of the Northern Hemisphere during MIS M2 in the otherwise globally warm early Late Pliocene. Numbers show sequence of events.

Our study identifies links between CAS through-flow, NAC variability, high latitude sea-surface temperatures, and Northern Hemisphere glaciation during Late Pliocene MIS M2 (∼3.30 Ma). We provide a conceptual model based on palynological and geochemical records in the North Atlantic and Caribbean for glacial expansion and consequent deglaciation during an otherwise globally warmer world (Figure 6).

A long-term global cooling trend (reflected in SST, CO_2_, and ice volume records; Figure 1) preconditioned the Northern Hemisphere for glaciation during the early Late Pliocene. However, the ultimate tipping point for intense glaciation during MIS M2 was the through-flow of Pacific water via an open CAS into the Atlantic, ultimately resulting in a steep SST gradient in the North Atlantic and thermal isolation of the high latitudes. An open CAS as the trigger for Northern Hemisphere glaciation contrasts with the usually invoked CAS closure as either the cause, precondition, or delaying factor for the intensification of Northern Hemisphere glaciation which occurred 500,000 years later [27], [33], [34]. Recent modelling experiments indicate that the closure of the CAS actually had no effect on the Late Pliocene Greenland ice sheet, and demonstrate that declining atmospheric carbon dioxide concentrations were the driving factor behind the intensification of Northern Hemisphere glaciation at ∼2.75 Ma [28], [71].

Our records in fact demonstrate that the glacio-eustatic closure of the CAS during MIS M2 eventually re-established northward heat transport in the North Atlantic. Following the expansion of the Antarctic and Northern Hemisphere ice sheets (including Greenland, Iceland, Svalbard/Barents region and questionably the interior of North America) to a volume seemingly larger than present, sea level fell to more than 10 m and possibly as much as 65 m below present (Figure 1B). This closed the CAS and halted the flow of Pacific water into the North Atlantic, allowing the Caribbean Warm Pool to accumulate. In time, this re-invigorated the Gulf Stream/North Atlantic Current system and provided northward heat transport, leading to high-latitude North Atlantic surface waters that were 3°C warmer than present and consequent retreat of the Greenland ice sheet to mountainous areas in the east and southeast during the mPWP.

The transition from MIS M2 to the mPWP can be seen as the evolution of a world with comparable global temperatures to present and slightly larger ice sheets, to a world with global temperatures ∼3°C higher than today and glaciation strongly diminished and localised in the Northern Hemisphere. Although operating on a longer time scale, this climate transition can provide valuable insights into the present anthropogenically-forced climate transition towards a globally warmer planet, being comparable to projections for the end of this century. In view of this projected climate warming, our results from the Late Pliocene show that high-latitude North Atlantic surface circulation and SSTs are a crucial factor in the expansion and contraction of Northern Hemisphere ice sheets.

## Supporting Information

Figure S1 **Age model for DSDP Hole 610A based on the correlation of oxygen isotope records from the studied intervals with the LR04 benthic oxygen isotope global stack** [**10**] **.** *Left panel*: core-sections, polarity subchrons, including uncertainty interval for the exact position of each reversal of the Mammoth Subchron, and benthic isotope record against depth (mbsf). *Middle panel*: correlation of the benthic record (thin red line, raw data; thick red line, 4-point running mean) to the LR04 global stack of benthic isotope records [10] plotted against time. Grey shading represents the marine isotope stage boundaries from [10]: marine isotope stage M2 was defined between 3.264 and 3.312 Ma. We consider the full glaciation to occur between 3.305 and 3.385 Ma (light grey). Thin black lines between left and middle panel show the tie points used (listed in inset). *Right panel*: sedimentation rate based on our age model. *Inset* gives the tie points used, and correlation values of the benthic record running mean and raw data with the LR04 global stack. Note: Hole 610A shows a coring gap between Cores 610A-17H and 610A-18H, and sediment disturbance in the upper 25 cm of Section 610A-18H1.
(TIF)

Figure S2 **Age model for IODP Site U1308 based on the correlation of oxygen isotope records from the studied intervals with the LR04 benthic oxygen isotope global stack** [**10**] **.** *Left, middle and right panel and inset* as for Figure S1.
(TIF)

Figure S3 **Age model for IODP Site U1313 based on the correlation of oxygen isotope records from the studied intervals with the LR04 benthic oxygen isotope global stack** [**10**] **.** *Left, middle and right panel and inset* as for Figure S1.
(TIF)

Figure S4 **Age model for DSDP Site 603 based on the correlation of oxygen isotope records from the studied intervals and palaeomagnetic reversals with the LR04 benthic oxygen isotope global stack** [**10**] **.** *Left, middle and right panel and inset* as for Figure S1.
(TIF)

Figure S5 **Shown on the left are the benthic δ^18^O global LR04 stack** [**10**] **compared to the benthic δ^18^O record of IODP Site 1241** [**69**] **.** On the right, the LR04 global stack is compared to the old [51] and new (this study) benthic δ^18^O curve of ODP Site 999. The latter is a fine-tuning of the [51] record to the LR04 stack.
(TIF)

## References

[1] Dowsett HJ, Robinson MM, Haywood AM, Hill DJ, Dolan AM, et al (2012) Assessing confidence in Pliocene sea surface temperatures to evaluate predictive models. Nat Clim Chang 2: 1–7 10.1038/nclimate1455

[2] Haywood AM, Valdes PJ (2004) Modelling Pliocene warmth: contribution of atmosphere, oceans and cryosphere. Earth Planet Sci Lett 218: 363–377 10.1016/S0012-821X(03)00685-X

[3] Raymo ME, Mitrovica JX, O'Leary MJ, Deconto RM, Hearty PJ (2011) Departures from eustasy in Pliocene sea-level records. Nat Geosci 4: 328–332 10.1038/ngeo1118

[4] Dolan AM, Haywood AM, Hill DJ, Dowsett HJ, Hunter SJ, et al (2011) Sensitivity of Pliocene ice sheets to orbital forcing. Palaeogeogr Palaeoclimatol Palaeoecol 309: 98–110 10.1016/j.palaeo.2011.03.030

[5] Zhang ZS, Nisancioglu KH, Chandler MA, Haywood AM, Otto-Bliesner BL, et al (2013) Mid-Pliocene Atlantic meridional overturning circulation not unlike modern. Clim Past 9: 1495–1504 10.5194/cp-9-1495-2013

[6] Raymo ME, Grant B, Horowitz M, Rau GH (1996) Mid-Pliocene warmth: stronger greenhouse and stronger conveyor. Mar Micropaleontol 27: 313–326 10.1016/0377-8398(95)00048-8

[7] Pagani M, Liu Z, LaRiviere J, Ravelo AC (2009) High Earth-system climate sensitivity determined from Pliocene carbon dioxide concentrations. Nat Geosci 3: 27–30 10.1038/ngeo724

[8] Seki O, Foster GL, Schmidt DN, Mackensen A, Kawamura K, et al (2010) Alkenone and boron-based Pliocene pCO_2_ records. Earth Planet Sci Lett 292: 201–211 10.1016/j.epsl.2010.01.037

[9] Bartoli G, Hönisch B, Zeebe RE (2011) Atmospheric CO_2_ decline during the Pliocene intensification of Northern Hemisphere glaciations. Paleoceanography 26: PA4213 10.1029/2010PA002055

[10] Lisiecki LE, Raymo ME (2005) A Pliocene-Pleistocene stack of 57 globally distributed benthic δ^18^O records. Paleoceanography 20: PA1003 10.1029/2004PA001071

[11] Mudelsee M, Raymo ME (2005) Slow dynamics of the Northern Hemisphere glaciation. Paleoceanography 20: PA4022 10.1029/2005PA001153

[12] Miller KG, Kominz MA, Browning JV, Wright JD, Mountain GS, et al (2005) The Phanerozoic record of global sea-level change. Science 310: 1293–1298 10.1126/science.1116412 16311326

[13] Naish TR, Wilson GS (2009) Constraints on the amplitude of Mid-Pliocene (3.6–2.4 Ma) eustatic sea-level fluctuations from the New Zealand shallow-marine sediment record. Phil Trans A Math Phys Eng Sci 367: 169–187 10.1098/rsta.2008.0223 18852088

[14] Miller KG, Wright JD, Browning JV, Kulpecz A, Kominz M, et al (2012) High tide of the warm Pliocene: Implications of global sea level for Antarctic deglaciation. Geology 40: 407–410 10.1130/G32869.1

[15] Dwyer GS, Chandler MA (2009) Mid-Pliocene sea level and continental ice volume based on coupled benthic Mg/Ca palaeotemperatures and oxygen isotopes. Phil Trans A Math Phys Eng Sci 367: 157–168 10.1098/rsta.2008.0222 18854304

[16] Brigham-Grette J, Melles M, Minyuk P, Andreev A, Tarasov P, et al.. (2013) Pliocene warmth, polar amplification, and stepped Pleistocene cooling recorded in NE Arctic Russia. Science 340, 1421–1427. doi:10.1126/science.1233137. 10.1126/science.1233137 23661643

[17] Pollard D, DeConto RM (2009) Modelling West Antarctic ice sheet growth and collapse through the past five million years. Nature 458: 329–333 10.1038/nature07809 19295608

[18] Naish T, Powell R, Levy R, Wilson G, Scherer R, et al (2009) Obliquity-paced Pliocene West Antarctic ice sheet oscillations. Nature 458: 322–328 10.1038/nature07867 19295607

[19] Passchier S (2011) Linkages between East Antarctic Ice Sheet extent and Southern Ocean temperatures based on a Pliocene high-resolution record of ice-rafted debris off Prydz Bay, East Antarctica. Paleoceanography 26: PA4204 10.1029/2010PA002061

[20] Kleiven HF, Jansen E, Fronval T, Smith TM (2002) Intensification of Northern Hemisphere glaciations in the circum Atlantic region (3.5–2.4 Ma) – ice-rafted detritus evidence. Palaeogeogr Palaeoclimatol Palaeoecol 184: 213–223 10.1016/S0031-0182(01)00407-2

[21] Knies J, Matthiessen J, Vogt C, Laberg JS (2009) The Plio-Pleistocene glaciation of the Barents Sea–Svalbard region: a new model based on revised chronostratigraphy. Quat Sci Rev 28: 812–829 10.1016/j.quascirev.2008.12.002

[22] Moran K, Backman J, Brinkhuis H, Clemens SC, Cronin T, et al (2006) The Cenozoic palaeoenvironment of the Arctic Ocean. Nature 441: 601–605 10.1038/nature04800 16738653

[23] Sarnthein M, Bartoli G, Prange M, Schmittner A, Schneider B, et al (2009) Mid-Pliocene shifts in ocean overturning circulation and the onset of Quaternary-style climates. Clim Past 5: 269–283 10.5194/cp-5-269-2009

[24] Geirsdóttir Á (2011) Pliocene and Pleistocene glaciations of Iceland: a brief overview of the glacial history. In: Ehlers J, Gibbard PL, Hughes PD, editors. Developments in Quaternary Science, Vol. 15. Amsterdam: Elsevier. 199–210. doi:10.1016/B978-0-444-53447-7.00016-7.

[25] Barendregt RW, Duk-Rodkin A (2011) Chronology and extent of Late Cenozoic ice sheets in North America: a magnetostratigraphical assessment. In: Ehlers J, Gibbard PL, Hughes PD, editors. Developments in Quaternary Science, Vol. 15. Amsterdam: Elsevier. 419–426. doi:10.1016/B978-0-444-53447-7.00032-5.

[26] De Schepper S, Head MJ, Groeneveld J (2009) North Atlantic Current variability through marine isotope stage M2 (circa 3.3 Ma) during the mid-Pliocene. Paleoceanography 24: PA4206 10.1029/2008PA001725

[27] Haug GH, Tiedemann R (1998) Effect of the formation of the Isthmus of Panama on Atlantic Ocean thermohaline circulation. Nature 393: 673–676 10.1038/31447

[28] Lunt DJ, Foster G, Haywood AM, Stone E (2008) Late Pliocene Greenland glaciation controlled by a decline in atmospheric CO_2_ levels. Nature 454: 1102–1106 10.1038/nature07223 18756254

[29] Brierley CM, Fedorov AV (2010) Relative importance of meridional and zonal sea surface temperature gradients for the onset of the ice ages and Pliocene–Pleistocene climate evolution. Paleoceanography 25: PA2214 10.1029/2009PA001809

[30] Naafs BDA, Stein R, Hefter J, Khélifi N, De Schepper S, et al (2010) Late Pliocene changes in the North Atlantic Current. Earth Planet Sci Lett 298: 434–442 10.1016/j.epsl.2010.08.023

[31] Lawrence KT, Sosdian S, White HE, Rosenthal Y (2010) North Atlantic climate evolution through the Plio-Pleistocene climate transitions. Earth Planet Sci Lett 300: 329–342 10.1016/j.epsl.2010.10.013

[32] Cane MA, Molnar P (2001) Closing of the Indonesian seaway as a precursor to east African aridification around 3–4| million years ago. Nature 411: 157–162 10.1038/35075500 11346785

[33] Driscoll NW, Haug GH (1998) A short circuit in thermohaline circulation: a cause for Northern Hemisphere glaciation? Science 282: 436–438 10.1126/science.282.5388.436 9774262

[34] Berger W, Wefer G (1996) Expeditions into the Past: Paleoceanographic studies in the South Atlantic. In Wefer G, Berger WH, Siedler G, Webb DJ, editors. The South Atlantic: Present and Past Circulation. Berlin, Heidelberg: Springer-Verlag. 363–410. doi: 10.1007/978-3-642-80353-6_21.

[35] De Schepper S, Fischer EI, Groeneveld J, Head MJ, Matthiessen J (2011) Deciphering the palaeoecology of Late Pliocene and Early Pleistocene dinoflagellate cysts. Palaeogeogr Palaeoclimatol Palaeoecol 309: 17–32 10.1016/j.palaeo.2011.04.020

[36] De Schepper S, Head MJ (2008) New dinoflagellate cyst and acritarch taxa from the Pliocene and Pleistocene of the eastern North Atlantic (DSDP Site 610). J Syst Paleont 6: 101–117 10.1017/S1477201907002167

[37] Stockmarr J (1971) Tablets with spores used in absolute pollen analysis. Pollen et Spores 13: 615–621.

[38] Heslop D, De Schepper S, Proske U (2011) Diagnosing the uncertainty of taxa relative abundances derived from count data. Mar Micropaleontol 79: 114–120 10.1016/j.marmicro.2011.01.007

[39] Harland R (1983) Distribution maps of Recent dinoflagellate cysts in bottom sediments from the North Atlantic Ocean and adjacent seas. Palaeontology 26: 321–387.

[40] Rochon A, de Vernal A, Turon J-L, Matthiessen J, Head MJ (1999) Distribution of recent dinoflagellate cysts in surface sediments from the North Atlantic Ocean and adjacent seas in relation to sea-surface parameters. AASP Contributions Series 35. Dallas, Texas: AASP Foundation. 146.

[41] Zonneveld KAF, Marret F, Versteegh GJM, Bogus K, Bonnet S, et al.. (2012) Atlas of modern dinoflagellate cyst distribution based on 2405 datapoints. Rev Palaebot Palynol. doi:10.1016/j.revpalbo.2012.08.003.

[42] Radi T, de Vernal A (2008) Dinocysts as proxy of primary productivity in mid–high latitudes of the Northern Hemisphere. Mar Micropaleontol 68: 84–114 10.1016/j.marmicro.2008.01.012

[43] Eynaud F, Turon J-L, Duprat J (2004) Comparison of the Holocene and Eemian palaeoenvironments in the South Icelandic Basin: dinoflagellate cysts as proxies for the North Atlantic surface circulation. Rev Palaebot Palynol 128: 55–79 10.1016/S0034-6667(03)00112-X

[44] Shackleton NJ, Hall MA (1984) Oxygen and carbon isotope stratigraphy of Deep Sea Drilling Project Hole 552A: Plio-Pleistocene glacial history. DSDP Init Rep 81: 599–609 10.2973/dsdp.proc.81.116.1984

[45] Barker S, Greaves M, Elderfield H (2003) A study of cleaning procedures used for foraminiferal Mg/Ca paleothermometry. Geochem Geophys Geosyst 4: 8407 10.1029/2003GC000559

[46] Greaves M, Caillon N, Rebaubier H, Bartoli G, Boharty S, et al (2008) Interlaboratory comparison study of calibration standards for foraminiferal Mg/Ca thermometry. Geochem Geophys Geosyst 9: Q08010 10.1029/2008GC001974

[47] Elderfield H, Ganssen G (2000) Past temperature and δ^18^O of surface ocean waters inferred from foraminiferal Mg/Ca ratios. Nature 405: 442–445 10.1038/35013033 10839536

[48] Schiebel R, Bijma J, Hemleben C (1997) Population dynamics of the planktic foraminifer *Globigerina bulloides* from the eastern North Atlantic. Deep Sea Res I 44(9–10): 1701–1713 10.1016/S0967-0637(97)00036-8

[49] Chapman MR (2010) Seasonal production patterns of planktonic foraminifera in the NE Atlantic Ocean: implications for paleotemperature and hydrographic reconstructions. Paleoceanography 25: PA1101 10.1029/2008PA001708

[50] Ganssen GM, Kroon D (2000) The isotopic signature of planktonic foraminifera from NE Atlantic surface sediments: implications for the reconstruction of past oceanic conditions. J Geol Soc Lond 157: 693–699 10.1144/jgs.157.3.693

[51] Steph S, Tiedemann R, Prange M, Groeneveld J, Nürnberg D, et al (2006) Changes in Caribbean surface hydrography during the Pliocene shoaling of the Central American Seaway. Paleoceanography 21: PA4221 10.1029/2008PA001645

[52] Nürnberg D, Müller A, Schneider RR (2010) Paleo-sea surface temperature calculations in the equatorial east Atlantic from Mg/Ca ratios in planktic foraminifera: A comparison to sea surface temperature estimates from *U* ^k′^ _37_, oxygen isotopes, and foraminiferal transfer function. Paleoceanography 15: 124–134 10.1029/1999PA000370

[53] Groeneveld J, Chiessi CM (2011) Mg/Ca of *Globorotalia inflata* as a recorder of permanent thermocline temperatures in the South Atlantic. Paleoceanography 26: PA2203 10.1029/2010PA001940

[54] Shackleton NJ (1974) Attainment of isotopic equilibrium between ocean water and benthonic foraminifera genus *Uvigerina*: Isotopic changes in the ocean during the last glacial. Colloq Int CNRS 219: 203–210.

[55] Naafs BDA, Hefter J, Acton G, Haug GH, Martinez-Garcia A, et al (2012) Strengthening of North American dust sources during the late Pliocene (2.7Ma). Earth Planet Sci Lett 317–318: 8–19 10.1016/j.epsl.2011.11.026

[56] Brassell SC, Eglington G, Marlowe IT, Pflaumann U, Sarnthein M (1986) Molecular stratigraphy: a new tool for climatic assessment. Nature 320: 129–133 10.1038/320129a0

[57] Prahl FG, Wakeham SG (1987) Calibration of unsaturation patterns in long-chain ketone compositions for paleotemperature assessment. Nature 330: 367–369 10.1038/330367a0

[58] Hefter J (2008) Analysis of alkenone unsaturation indices with fast gas chromatography/time-of-flight mass spectrometry. Anal Chem 80: 2161–2170 10.1021/ac702194m 18288817

[59] Müller PJ, Kirst G, Ruhland G, von Storch I, Rosell-Melé A (1998) Calibration of the alkenone paleotemperature index *U* ^k′^ _37_ based on core-tops from the eastern South Atlantic and the global ocean (60°N–60°S). Geochim Cosmochem Acta 62: 1757–1772 10.1016/S0016-7037(98)00097-0

[60] Schiebel R, Brupbacher U, Schmidtko S, Nausch G, Waniek JJ, et al (2011) Spring coccolithophore production and dispersion in the temperate eastern North Atlantic Ocean. J Geophys Res 116: C08030 10.1029/2010JC006841

[61] Clement BM, Robinson F (1987) The magnetostratigraphy of Leg 94 sediments. DSDP Init Rep 94: 635–650 10.2973/dsdp.proc.94.112.1987

[62] Expedition 303 Scientists (2006) Site U1308. Proc IODP 303/306: 1–98 10.2204/iodp.proc.303306.108.2006

[63] Lourens L, Hilgen F, Shackleton NJ, Laskar J, Wilson D (2005) The Neogene. In: Gradstein FM, Ogg JG, Smith AG, editors. A Geological Time Scale 2004. Cambridge: Cambridge University Press. 409–430. doi: 10.1017/CBO9780511536045

[64] Shipboard Scientific Party (1987) Site 610. DSDP Init Rep 94: 351–470 10.2973/dsdp.proc.94.106.1987

[65] Expedition 306 Scientists (2006) Site U1313. Proc IODP 303/306: 1–124 10.2204/iodp.proc.303306.112.2006

[66] Paillard D, Labeyrie L, Yiou P (1996) Macintosh Program performs time-series analysis. Eos Trans AGU 77: 379 10.1029/96EO00259

[67] Shackleton NJ, Hall MA, Pate D, Pisias N, Mayer L, et al (1995) Pliocene stable isotope stratigraphy of ODP Site 846. Proc ODP Sci Res 138: 337–356 10.2973/odp.proc.sr.138.117.1995

[68] Tiedemann R, Sarnthein M, Shackleton NJ (1994) Astronomic timescale for the Pliocene Atlantic δ^18^O and dust flux records of Ocean Drilling Program Site 659. Paleoceanography 9: 619–638 10.1029/94PA00208

[69] Tiedemann R, Sturm A, Steph S, Lund SP, Stoner JS, et al (2006) Astronomically calibrated timescales from 6 to 2.5 Ma and benthic isotope stratigraphies, Sites 1236, 1237, 1239, and 1241. Proc ODP Sci Res 202: 1–69 10.2973/odp.proc.sr.202.210.2007

[70] Schneider B, Schmittner A (2006) Simulating the impact of the Panamanian seaway closure on ocean circulation, marine productivity and nutrient cycling. Earth Planet Sci Lett 246: 367–380 10.1016/j.epsl.2006.04.028

[71] Lunt DJ, Valdes PJ, Haywood AM, Rutt IC (2008) Closure of the Panama Seaway during the Pliocene: implications for climate and Northern Hemisphere glaciation. Clim Dyn 30: 1–18 10.1007/s00382-007-0265-6

[72] Klocker A, Prange M, Schulz M (2005) Testing the influence of the Central American Seaway on orbitally forced Northern Hemisphere glaciation. Geophys Res Lett 32: L03703 10.1029/2004gl021564

[73] Darling KF, Kucera M, Wade CM, von Langen P, Pak D (2003) Seasonal distribution of genetic types of planktonic foraminifer morphospecies in the Santa Barbara Channel and its paleoceanographic implications. Paleoceanography 18(2): 1032 10.1029/2001PA000723

[74] Born A, Kageyama M, Nisancioglu KH (2010) Warm Nordic Seas delayed glacial inception in Scandinavia. Clim Past 6: 817–826 10.5194/cp-6-817-2010

[75] Salzmann U, Haywood AM, Lunt DJ, Valdes PJ, Hill DJ (2008) A new global biome reconstruction and data-model comparison for the Middle Pliocene. Glob Ecol Biogeogr 17: 432–447 10.1111/j.1466-8238.2008.00381.x

[76] Gao C, McAndrews JH, Wang X, Menzies J, Turton CL, et al (2012) Glaciation of North America in the James Bay Lowland, Canada, 3.5 Ma. Geology 40: 975–978 10.1130/G33092.1

[77] Carlson AE, Oppo DW, Came RE, Legrande AN, Keigwin LD, et al (2008) Subtropical Atlantic salinity variability and Atlantic meridional circulation during the last deglaciation. Geology 36: 991–994 10.1130/G25080A.1

[78] Steph S, Tiedemann R, Groeneveld J, Sturm A, Nürnberg D (2006) Pliocene changes in tropical East Pacific upper ocean stratification: response to tropical gateways? Proc ODP Sci Res 202: 1–51 10.2973/odp.proc.sr.202.211.2006

[79] Bintanja R, van de Wal RSW (2008) North American ice-sheet dynamics and the onset of 100,000-year glacial cycles. Nature 454: 869–872 10.1038/nature07158 18704083

[80] Kürschner W, van der Burgh J, Visscher H, Dilcher D (1996) Oak leaves as biosensors of late Neogene and early Pleistocene paleoatmospheric CO_2_ concentrations. Mar Micropaleontol 27: 299–312.

[81] Locarnini RA, Mishonov AV, Antonov JI, Boyer TP, Garcia HE (2006) World Ocean Atlas 2005, Volume 1: Temperature. In: Levitus S, editor. NOAA Atlas NESDIS 61. Washington, D.C.: U.S. Gov. Printing Office. p. 182. Available: http://www.nodc.noaa.gov/OC5/indprod.html.

