# Supplementary figures and images for: Northern Hemisphere Glaciation during the Globally Warm Early Late Pliocene
Source: PLoS One. 2013 Dec 12;8(12):e81508. doi: 10.1371/journal.pone.0081508 (PMC3861316; doi:10.1371/journal.pone.0081508)

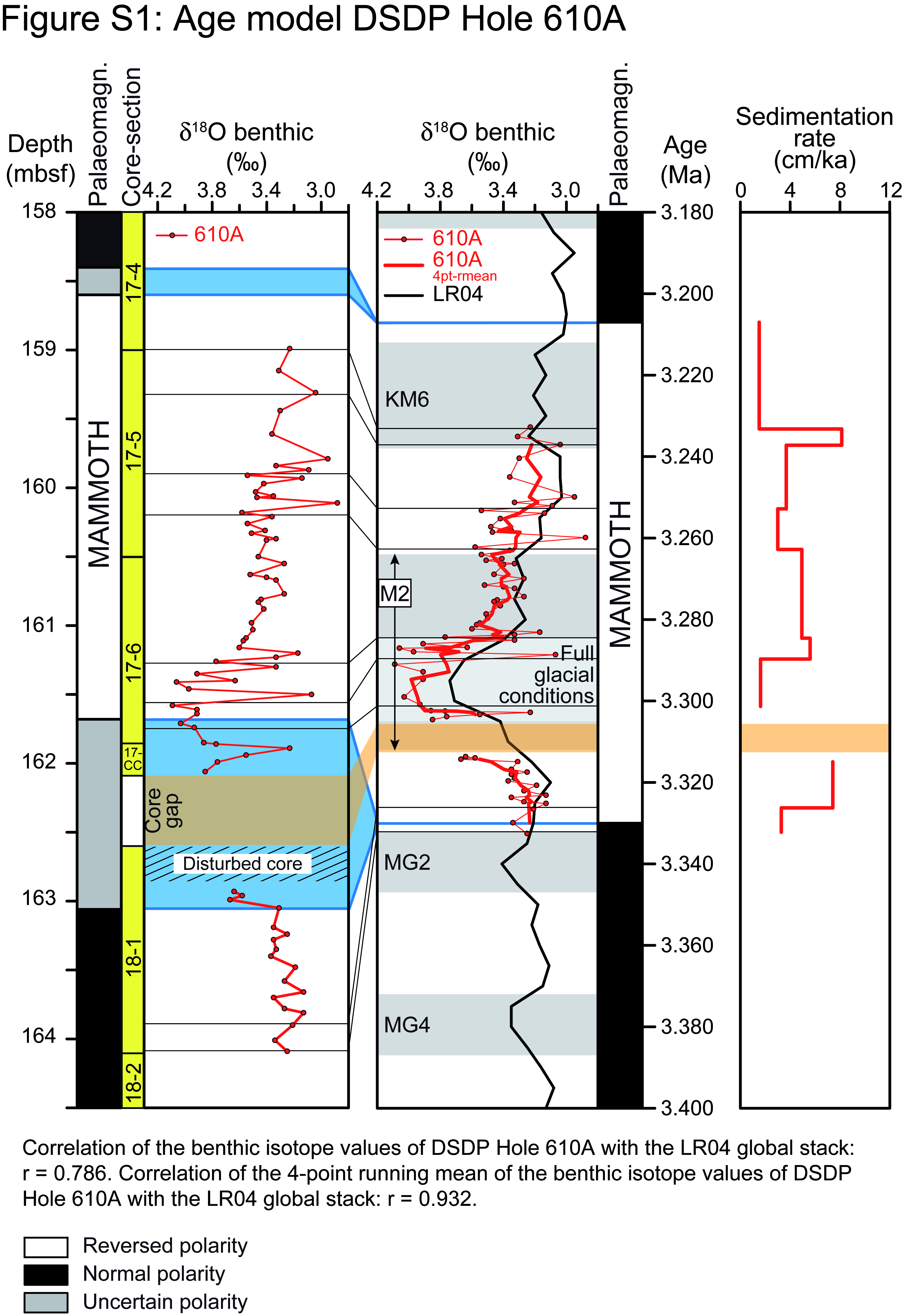

Supplement: Figure S1 — Age model for DSDP Hole 610A based on the correlation of oxygen isotope records from the studied intervals with the LR04 benthic oxygen isotope global stack [10] . Left panel: core-sections, polarity subchrons, including uncertainty interval for the exact position of each reversal of the Mammoth Subchron, and benthic isotope record against depth (mbsf). Middle panel: correlation of the benthic record (thin red line, raw data; thick red line, 4-point running mean) to the LR04 global stack of benthic isotope records [10] plotted against time. Grey shading represents the marine isotope stage boundaries from [10]: marine isotope stage M2 was defined between 3.264 and 3.312 Ma. We consider the full glaciation to occur between 3.305 and 3.385 Ma (light grey). Thin black lines between left and middle panel show the tie points used (listed in inset). Right panel: sedimentation rate based on our age model. Inset gives the tie points used, and correlation values of the benthic record running mean and raw data with the LR04 global stack. Note: Hole 610A shows a coring gap between Cores 610A-17H and 610A-18H, and sediment disturbance in the upper 25 cm of Section 610A-18H1. (TIF) [file pone.0081508.s001.tif]

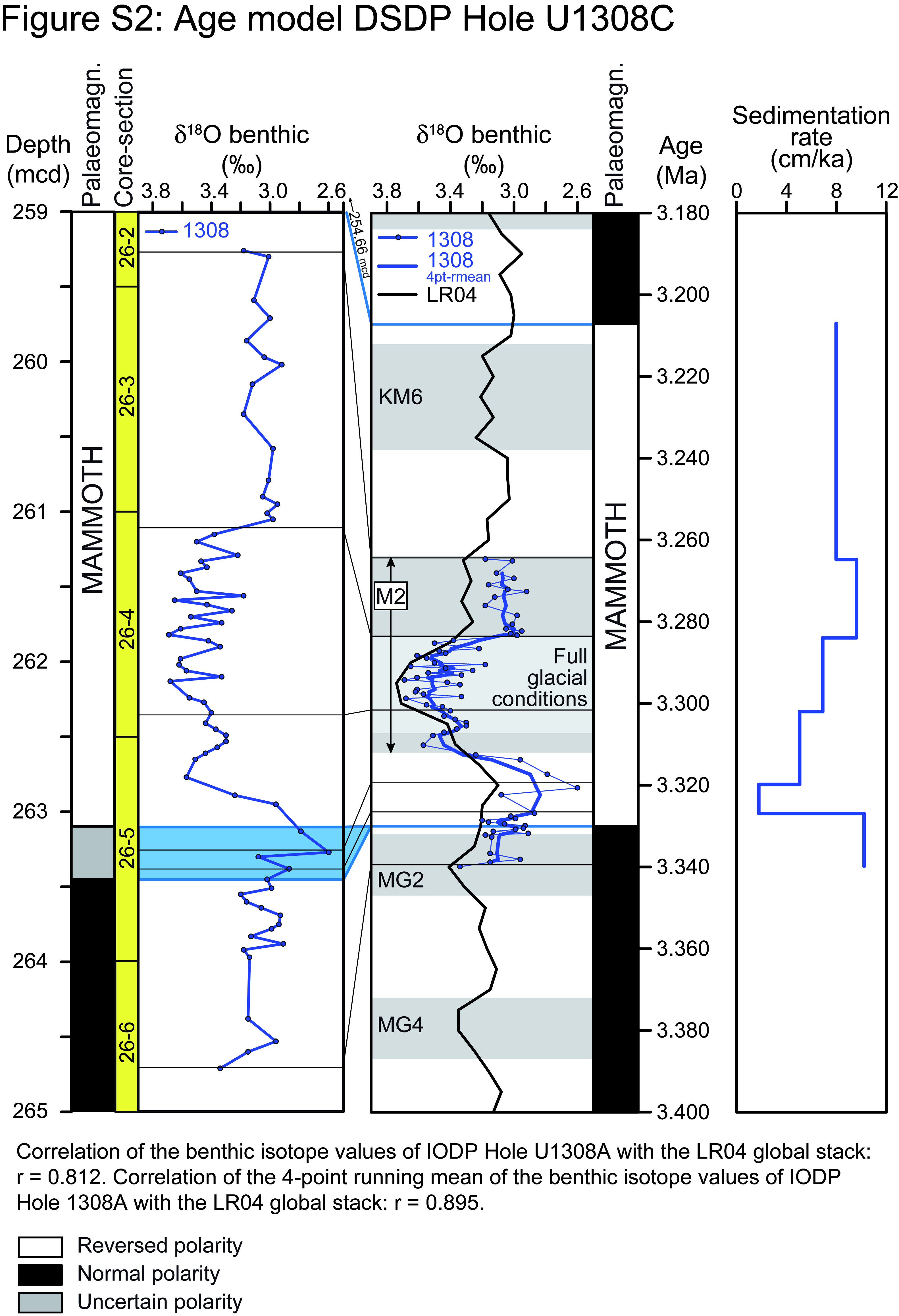

Supplement: Figure S2 — Age model for IODP Site U1308 based on the correlation of oxygen isotope records from the studied intervals with the LR04 benthic oxygen isotope global stack [10] . Left, middle and right panel and inset as for Figure S1. (TIF) [file pone.0081508.s002.tif]

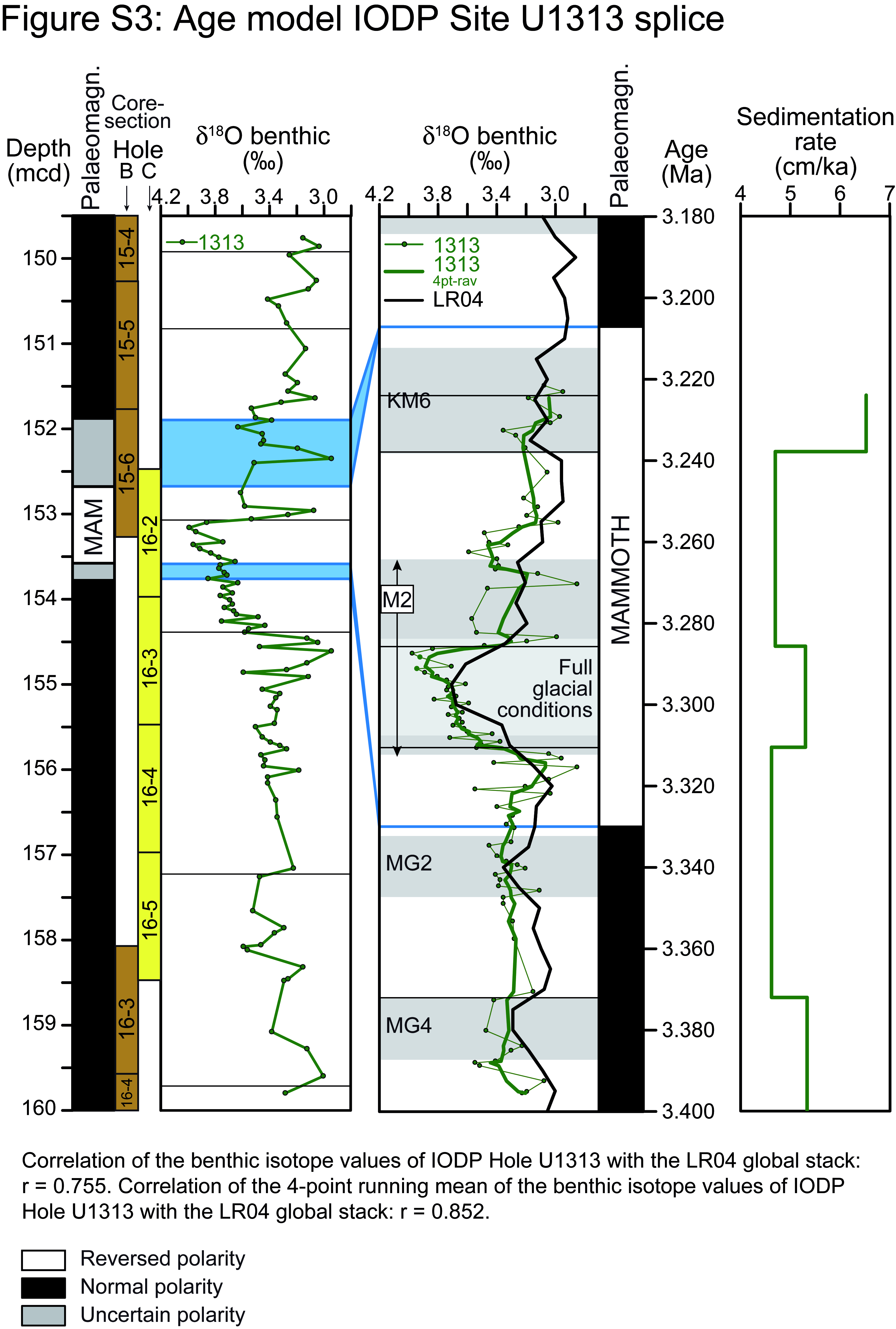

Supplement: Figure S3 — Age model for IODP Site U1313 based on the correlation of oxygen isotope records from the studied intervals with the LR04 benthic oxygen isotope global stack [10] . Left, middle and right panel and inset as for Figure S1. (TIF) [file pone.0081508.s003.tif]

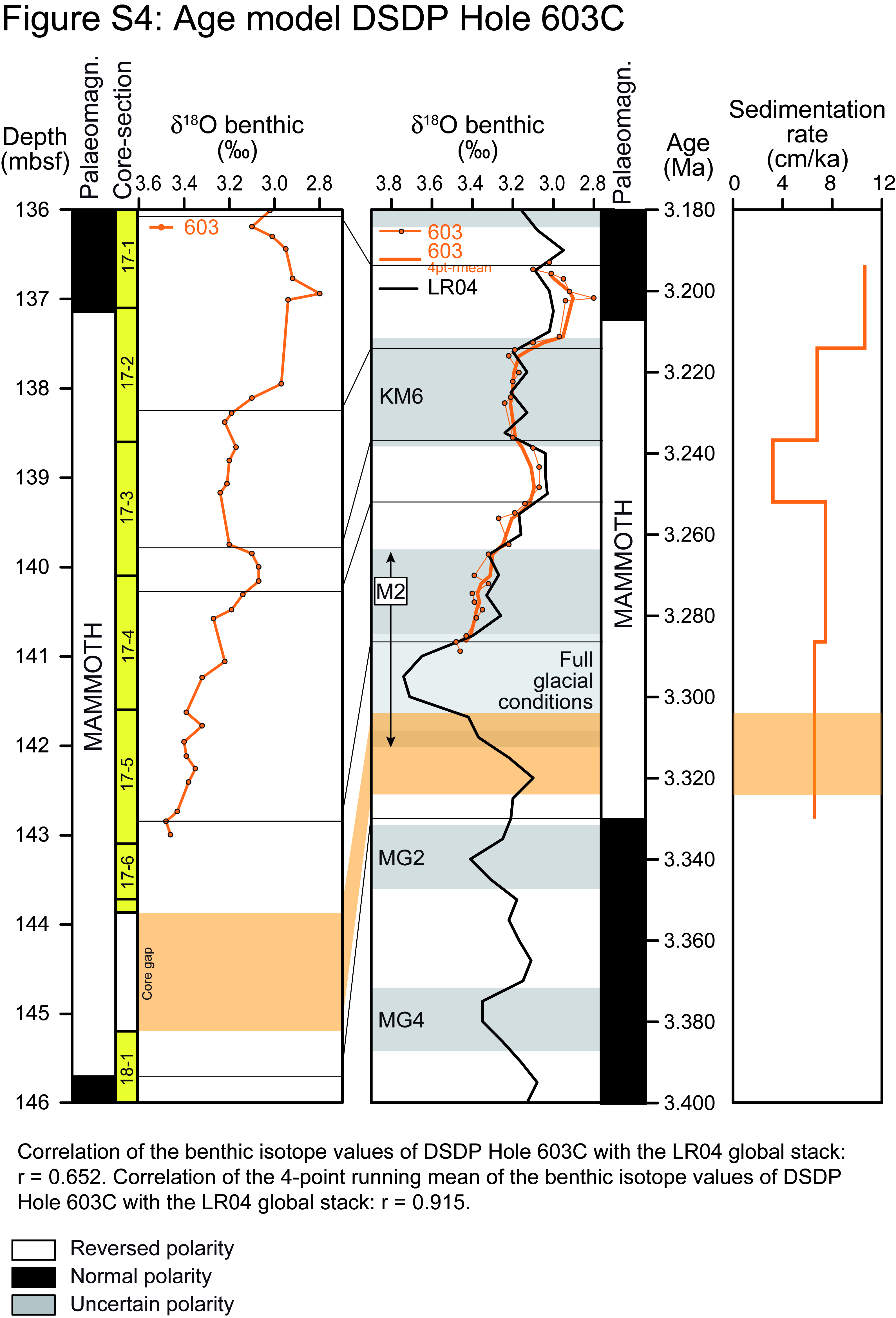

Supplement: Figure S4 — Age model for DSDP Site 603 based on the correlation of oxygen isotope records from the studied intervals and palaeomagnetic reversals with the LR04 benthic oxygen isotope global stack [10] . Left, middle and right panel and inset as for Figure S1. (TIF) [file pone.0081508.s004.tif]

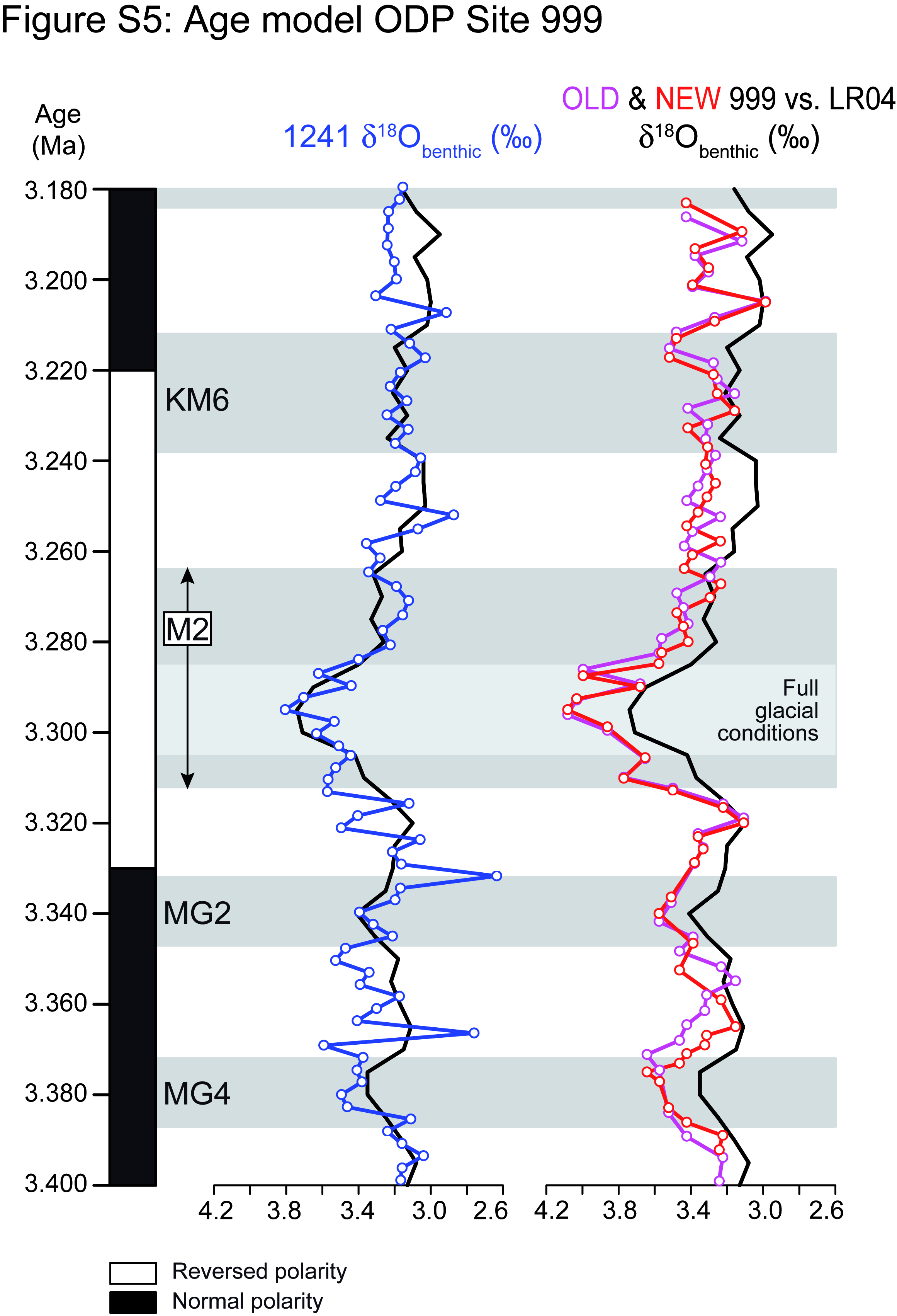

Supplement: Figure S5 — Shown on the left are the benthic δ18O global LR04 stack [10] compared to the benthic δ18O record of IODP Site 1241 [69] . On the right, the LR04 global stack is compared to the old [51] and new (this study) benthic δ18O curve of ODP Site 999. The latter is a fine-tuning of the [51] record to the LR04 stack. (TIF) [file pone.0081508.s005.tif]
